# An international, Delphi consensus study to identify priorities for methodological research in behavioral trials in health research

**DOI:** 10.1186/s13063-020-04235-z

**Published:** 2020-03-23

**Authors:** Molly Byrne, Jenny McSharry, Oonagh Meade, Kim L. Lavoie, Simon L. Bacon

**Affiliations:** 1 Health Behavior Change Research Group, School of Psychology, National University of Ireland, Galway, H91 TK33 Ireland; 2 Department of Psychology, University of Quebec at Montreal, Montreal, QC Canada; 3 Montreal Behavioral Medicine Centre, CIUSSS-NIM – Hôpital du Sacre-Coeur de Montreal, Montreal, QC Canada; 4 Department of Health, Kinesiology, and Applied Physiology, Concordia University, Montreal, QC H4B 1R6 Canada

**Keywords:** Behavior change interventions, Research prioritization, Randomized controlled trials, Methodological research, Delphi study

## Abstract

**Background:**

Non-communicable chronic diseases are linked to behavioral risk factors (including smoking, poor diet and physical inactivity), so effective behavior change interventions are needed to improve population health. However, uptake and impact of these interventions is limited by methodological challenges. We aimed to identify and achieve consensus on priorities for methodological research in behavioral trials in health research among an international behavioral science community.

**Methods:**

An international, Delphi consensus study was conducted. Fifteen core members of the International Behavioral Trials Network (IBTN) were invited to generate methodological items that they consider important. From these, the research team agreed a “long-list” of unique items. Two online surveys were administered to IBTN members (*N* = 306). Respondents rated the importance of items on a 9-point scale, and ranked their “top-five” priorities. In the second survey, respondents received feedback on others’ responses, before rerating items and re-selecting their top five.

**Results:**

Nine experts generated 144 items, which were condensed to a long-list of 33 items. The four most highly endorsed items, in both surveys 1 (*n* = 77) and 2 (*n* = 57), came from two thematic categories:“Intervention development” (“Specifying intervention components” and “Tailoring interventions to specific populations and contexts”) and “Implementation” (“How to disseminate behavioral trial research findings to increase implementation” and “Methods for ensuring that behavioral interventions are implementable into practice and policy”). “Development of novel research designs to test behavioral interventions” also emerged as a highly ranked research priority.

**Conclusions:**

From a wide array of identified methodological issues, intervention development, implementation and novel research designs are key themes to drive the future behavioral trials’ research agenda. Funding bodies should prioritize these issues in resource allocation.

## Introduction

Rapidly increasing rates of chronic disease are a key global societal challenge [51]. The leading behavioral risk factors are similar across chronic diseases including tobacco use, harmful alcohol consumption, unhealthy diet including high salt and sodium intake, physical inactivity, and being overweight and obesity [45]. Effective, evidence-based behavior change interventions are urgently needed to reduce the prevalence of chronic disease internationally and the burden these conditions place on patients and health services.

For the purposes of this study, behavioral interventions were defined as: “interventions that require the active participation of a target group (e.g., the patient/individual, health professional, health care systems) with the proximal or ultimate goal of changing health-related behavior.” Behavioral interventions may be delivered in person or digitally, employing digital technologies such as the Internet, telephones and mobile and environmental sensors [23]. Interventions may also be delivered as national campaigns, or through communities.

Within behavioral medicine, much research is focused on developing behavior change interventions to reduce chronic disease prevalence, mortality, and burden of disease [24]. However, despite the significant potential to improve health and clinical outcomes, the reach and impact of behavioral interventions remains limited [35]. Suboptimal behavior change research not only reduces the likelihood that this research impacts on health outcomes, but it is also cost-ineffective. In 2010, expenditure on life sciences (mostly biomedical) research internationally was US$240 billion [47]. Waste across medical research (clinical or other types) has been estimated as consuming 85% of the billions spent each year [25] and commentators have criticized clinical research suggesting that most research is not useful [18].

An array of reasons has been suggested for the limited success in behavior change research including: low investment in this area of research [33], poor quality evaluation methods [13], lack of application of behavior change theory [29], poor specification of intervention content [30] and lack of an interdisciplinary team science approach [12]. Behavior change intervention research involves development, testing and implementation of “complex” interventions, with multiple components and involving multiple stakeholders [8]. This type of research requires a more complex, biopsychosocial approach to evidence generation than has been previously applied to answering questions about the effectiveness of clinical interventions [48]. Behaviour change research raises unique methodological challenges for the researcher, which need to be addressed and overcome if we are to develop a strong evidence base for behavior change interventions.

The International Behavioral Trial Network (IBTN; www.ibtnetwork.org) was established by a team of behavioral researchers in June 2013 to address methodological challenges specifically relevant to behavioral trials’ research. The IBTN is a global network of professionals working to improve the quality of clinical trials and behavioral interventions, with three main goals: first, to facilitate the global improvement of the quality of behavioral trials; second, to create networks and capacity to undertake more and higher-quality trials; and third, to develop a repository of resources of existing recommendations, tools and methodology papers on behavioral trials and intervention development. Currently (June 2019), the IBTN has 322 members, from 30 different countries across the world, and includes academics/researchers, postgraduate students, health professionals, general public and industry representatives.

Improving the quality and potential of behavioral trials requires methodological issues in this area to be identified and research to be conducted with the specific aim of addressing these issues. Previously discussed methodological issues specific to the design and conduct of behavioral trials include intervention development and piloting, intervention reporting, identifying suitable comparison groups, selection of appropriate outcome measures and intervention fidelity [3]. However, a formal, systematic process to identify and specify methodological priorities is now needed to facilitate the development of an international and cohesive behavioral trials’ research agenda.

Research prioritization provides such a process, whereby key stakeholders generate ideas and move towards consensus on important research topics [43]. The prioritization process has been used to identify priorities across conditions and populations [26]. In the area of trials’ research prioritization has been conducted with Directors of UK Clinical Research Collaboration Clinical Trials Units to inform the broader trials’ methodological research agenda [49] and, more recently, a priority setting exercise has been reported to inform the global health trials’ methodology research agenda [46]. Research prioritization can provide useful information to guide research funders.

The aim of this study was to identify priorities for, and achieve consensus on, methodological research in behavioral trials in health research. This information is needed to inform and guide the direction of the behavioral trials’ research agenda internationally. This study used a Delphi priority-setting consensus approach, inviting all members of the IBTN to participate.

## Methods

The study protocol has been published elsewhere [5]. This Delphi study was conducted and is reported following the reporting standard for Conducting and REporting of DElphi Studies (CREDES) [21].

### The Delphi process

An electronic Delphi (e-Delphi), with online administration of questionnaires, was used for this research prioritization to facilitate international participation [10]. The Delphi process is a structured group facilitation technique to obtain consensus among anonymous respondents through iterative rounds with feedback [28]. The Delphi approach has been widely used in health research [20, 21]. The features of the Delphi process which make it suitable for gaining consensus include: anonymity to facilitate balanced participation and iterative rounds to allow participants to change their opinion in response to controlled feedback where participants are provided with information on the distribution of overall group responses from previous rounds [20].

### Participants

Participants for Phase 1, the topic generation phase, were 15 experts in behavioral trials selected by the research team. Experts included founding members of IBTN, members of the IBTN Executive Committee and members of the research team. All experts had a minimum of 10 years’ experience of behavioral trials and a reputation for leadership in the field. Participants for Phase 2, the e-Delphi survey, were all those registered as members of the IBTN in February 2018 (*N* = 306, including members from five continents).

### Delphi stages

**Fig. 1:**
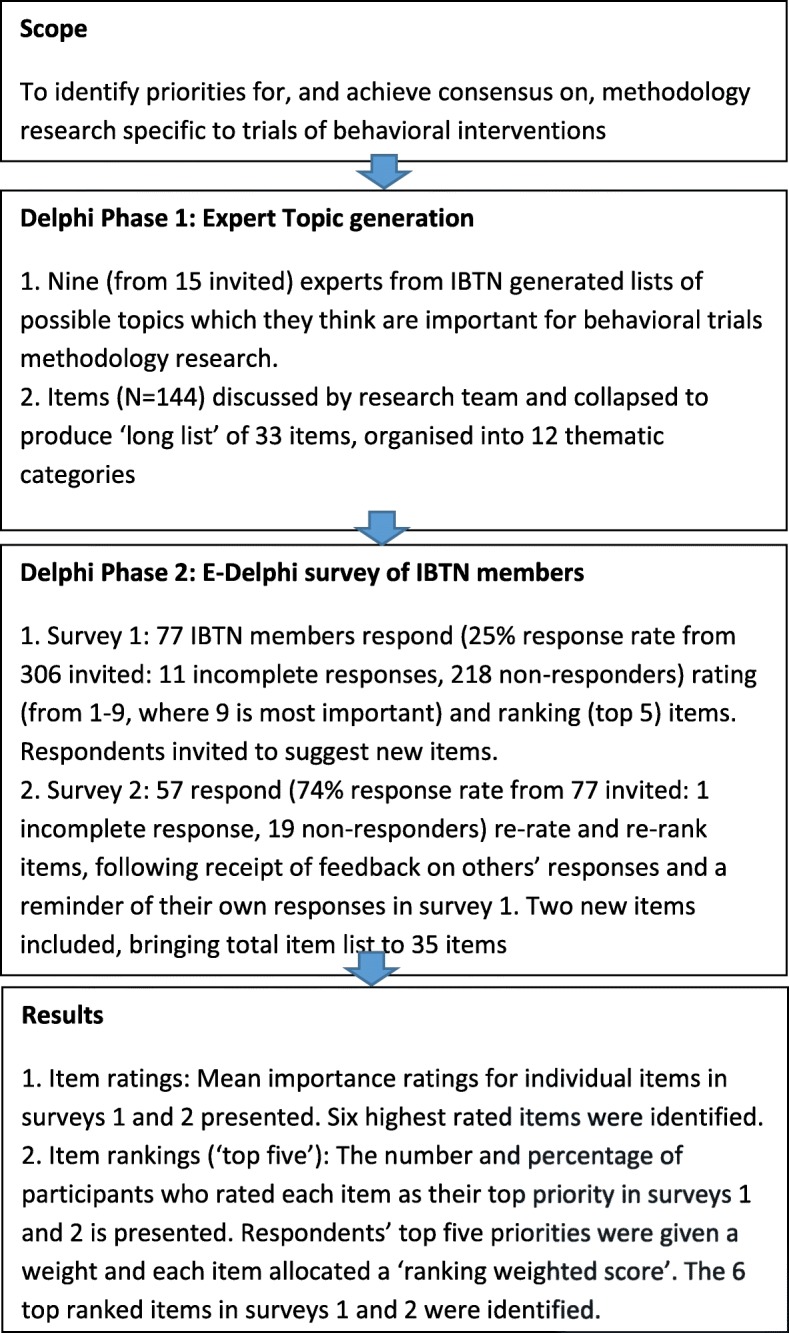
Flow chart to illustrate the stages of the Delphi process

See the flow chart in Fig. 1 which illustrates the stages of the Delphi process.

### Delphi Phase 1: expert topic generation

Fifteen experts in behavioral trials were contacted by a member of the research team (MB) by email in May 2017 and invited to generate a list of all possible topics or research questions which they consider important for behavioral trials’ methodology research. Respondents were asked to provide demographic information including: sex, current professional position, country of residence and number of years of experience of working in the area of trials of behavioral interventions.

Two members of the research team (MB and JMS) reviewed generated items initially, removing duplicates and merging similar topics, and along with two other members of the research team (KL and SB) agreed a draft “long-list” of unique items. This list was emailed to respondents to check for agreement and to see if items were faithful to the originally generated items, and feedback was discussed by the research team. The final long-list was approved and agreed by the research team in July 2017.

### Delphi Phase 2: E-Delphi survey

All members of the IBTN were invited by email to participate in two online surveys, using LimeSurvey online survey software (LimeSurvey GmbH, Hamburg, Germany. URL http://www.limesurvey.org).

The first survey was emailed to IBTN members (*N* = 306) in February 2018. Recipients were asked for their views on priorities for methodological research in trials of behavioral interventions. They were asked to rate the importance of each item on a 9-point scale, where 9 indicated items of highest importance and 1 indicated lowest importance. Following rating of the 33 items, they were asked to select and rank their “top-five” most important methodological research topics for trials of behavioral interventions. Respondents were provided with an open text-box to add any items which they believed were important and were missing from the list. Respondents were asked to provide demographic information including: sex, current professional position, country of residence, age group and number of years of experience of working in the area of trials of behavioral interventions.

In the second survey (administered 3 weeks after the closing of survey 1), participants who had responded to survey 1 received information reminding them of how they had responded in survey 1 and information about how others rated and ranked the items in survey 1. For rating the importance of individual items, bar charts plotting group responses to each item were provided, as well as the group mean importance rating for each item, and the individual’s own importance rating from survey 1. Respondents were asked to re-rate items with this information in mind. For the top-five ranking question, participants were reminded of their top-five selection from survey 1, and were presented with the percentage of respondents who had ranked each item in their top five in survey 1. Participants were asked to re-rank their top-five priority items with this information in mind.

Any additional items proposed in the free-text comment box in survey 1 were discussed by the research team and included for rating in survey 2 if the majority of team members agreed that the item was a unique, novel, previously excluded item. New items added to survey 2 were, therefore, rated only once in the Delphi process.

To encourage participation, the names of respondents to both surveys were entered into a draw for two prizes (personal fitness tracking devices). Only those who had responded to both surveys were included in the draw.

All data were extracted from the online survey software and imported into an SPSS database, which was stored anonymously on password-protected computers to which only members of the research team had access. Survey 2 ranked priority items were allocated a “ranking weighted score,” as follows: first priority was given 5; second priority was given 4; third priority was given 3; fourth priority was given 2; and fifth priority was given 1.

### Ethical approval

Ethical approval was granted by the National University of Ireland Galway Research Ethics Committee (reference: 17-Jun-13).

## Results

### Delphi Phase 1: expert topic generation

Nine of the 15 experts contacted agreed to participate and returned a list of items, representing a response rate of 60%. Of these, four were women and five were men. They were working in Canada, the UK, the US, Ireland and France, and had between 10 and 35 years of experience working in the area of behavioral interventions. Four of these nine experts were members of the research team; no other conflicts of interest related to research were disclosed by included experts.

In total, the nine experts generated 144 items. Following the initial review (by MB and JMS), removing duplicates and merging similar topics, the list was reduced to 40 items, which were organized for ease of review and to aid comprehension into 12 categorical themes. The categorical themes, agreed by the research team, were: Intervention Development; Comparison Group; Intervention Fidelity; Pilot/Feasibility trials; Reporting; Novel Trial Designs; Data Issues; Outcomes; Cost-effectiveness; Implementation; Stakeholder engagement; and Development of behavioral science and theory. Following feedback from the experts and discussions among the research team, the final list for survey 1 included 33 items, organized into the same 12 categorical themes. The list can be seen in Table 1.

**Table 1:** The “long-list” of items for methodological research in trials of behavioral interventions agreed in Phase 1

Categories	Item
**Intervention Development**	1. Using theory in behavioral intervention development
	2. Use of systematic approaches to move from evidence to intervention components
	3. Specifying intervention components
	4. Exploring impact of mode of intervention delivery
	5. Tailoring interventions to specific populations and contexts
**Comparison Group**	6. Selection of suitable comparison group(s) within trials
	7. Contamination between study arms (intervention and comparison) within trials
	8. Blinding of researchers and participants to study-arm allocation
**Intervention Fidelity**	9. Impact on intervention delivery of characteristics (such as qualifications and training) of those delivering interventions
	10. Strategies to optimize intervention fidelity
	11. Methods to assess intervention fidelity
	12. Strategies to maximize trial participant recruitment and retention
**Pilot/Feasibility trials**	13. Establishing criteria for progressing from trial piloting phases to full randomized controlled trial (RCT)
	14. Sample-size calculations for pilot trials
	15. Novel approaches and designs for piloting behavioral interventions
**Reporting**	16. Standardizing methods for reporting behavioral trials
	17. Reporting intervention and comparison group(s) intervention content
	18. Standardized methods for reporting and registering behavioral trials’ protocols
**Novel Trial Designs**	19. Development of novel research designs to test behavioral interventions as alternatives to, or to complement, standard RCTs
**Data Issues**	20. Strategies for handling missing data within behavioral trials
	21. Developing novel statistical techniques to enhance behavioral trials
**Outcomes**	22. Determining clinically significant changes in outcomes within trials
	23. Selecting appropriate behavioral outcomes for trials
	24. Relationship between behavioral outcomes and clinical/other outcomes
	25. Determining ideal timing of outcome measurement within trials
	26. Measurement of process(es) of change or mechanisms of action within interventions
**Cost-effectiveness**	27. Methods for cost-effectiveness analyses for behavioral trials
**Implementation**	28. Methods for ensuring that behavioral interventions are implementable into practice and policy
	29. How to disseminate behavioral trial research findings to increase implementation
**Stakeholder engagement**	30. How to optimize stakeholder engagement in behavioral trial research
	31. Incorporating stakeholder input in intervention development and delivery
	32. Testing the impact of stakeholder engagement in behavioral trial research
**Development of behavioral science and theory**	33. Trials’ research to test and develop behavioral theories

### Delphi Phase 2: E-Delphi survey

Response rates: of the 306 invitations sent in survey 1, complete responses were received from 77 people (25% response rate); incomplete responses were returned from 11 people and 218 people did not respond. Of the 77 invitations sent in survey 2, complete responses were received from 57 people (74% response rate); incomplete responses were returned from one person and 19 people did not respond. Only complete responses were used in the analysis. The professional background and demographic data for survey-1 and -2 completers are shown in Table 2. In survey 1, 69% of respondents were female. The majority (64%) had academic positions, 22% were students (undergraduate and graduate) and the remainder were health care practitioners, policy-makers or described themselves as “other.” Forty-three percent lived in Canada, 16% the US, 16% in Ireland, with of the remaining 25% of participants living in: Israel, Australia, Netherlands, Portugal, Sweden, UK, Brazil, China, Columbia and France. The majority of respondents were between the ages of 31 and 50 years (58%). Thirty-five percent of respondents had between 1 and 5 years’ experience in behavioral trials’ research, but it is worth noting that 26% reported having more than 10 years’ experience.

**Table 2:** Professional background and demographic data for survey completers

	Survey round 1 *n* = 77	Survey round 2 *n* = 57
Gender		
Male	23 (29.9%)	17 (29.8%)
Female	53 (68.8%)	39 (68.4%)
Other	1 (1.3%)	1 (1.8%)
Professional position		
University student (undergraduate/postgraduate)	17 (22.1%)	12 (21.1%)
Academic staff (e.g., researchers, lecturers, professors)	49 (63.6%)	38 (66.7%)
Health care practitioner	2 (2.6%)	1 (1.8%)
Health policy-maker or planner	2 (2.6%)	1 (1.8%)
Other	7 (9.1%)	5 (8.8%)
Country of residence (in alphabetical order)		
Australia	2 (2.6%)	1 (1.8%)
Brazil	1 (1.3%)	1 (1.8%)
Canada	33 (42.9%)	25 (43.9%)
China	1 (1.3%)	1 (1.8%)
Columbia	1 (1.3%)	1 (1.8%)
France	4 (5.2%)	2 (3.5%)
Ireland	12 (15.6%)	8 (14.0%)
Israel	1 (1.3%)	1 (1.8%)
Netherlands	1 (1.3%)	1 (1.8%)
Portugal	1 (1.3%)	1 (1.8%)
Sweden	1 (1.3%)	1 (1.8%)
UK	7 (9.1%)	5 (8.8%)
USA	12 (15.6%)	9 (15.8%)
Age group		
18–30 years	18 (23.4%)	14 (24.6%)
31–40 years	28 (36.4%)	19 (33.3%)
41–50 years	17 (22.1%)	11 (19.3%)
51 + years	14 (18.2%)	13 (22.8%)
Years of experience in trials of behavioral interventions		
Less than 1 year	12 (15.6%)	8 (14.0%)
1–5 years	27 (35.1%)	19 (33.3%)
6–10 years	18 (23.4%)	14 (24.6%)
10 + years	20 (26.0%)	16 (28.1%)

The mean importance ratings for individual items in surveys 1 and 2 can be seen in Table 3. The same six items were the six most highly rated items in both surveys 1 and 2, although the order changed slightly. These were (in order of descending levels of importance from the most highly rated item from the ratings in survey 2): Specifying intervention components; How to disseminate behavioral trial research findings to increase implementation; Methods for ensuring that behavioral interventions are implementable into practice and policy; Use of systematic approaches to move from evidence to intervention components; Selecting appropriate behavioral outcomes for trials; and Tailoring interventions to specific populations and contexts. The four most highly rated items, in both surveys 1 and 2, came from two of the categories: Intervention development (Specifying intervention components and Use of systematic approaches to move from evidence to intervention components) and Implementation (How to disseminate behavioral trial research findings to increase implementation, and Methods for ensuring that behavioral interventions are implementable into practice and policy).

**Table 3:** Mean importance ratings for individual items in surveys 1 and 2, ordered by survey 2 importance ratings (possible score range 1–9: 1 = lowest importance, 9 = highest importance)

Research items	Survey 1			Survey 2		
	Mean	SD	Rank	Mean	SD	Rank
1. Specifying intervention components	7.81	1.31	3	8.33	.81	1
2. How to disseminate behavioral trial research findings to increase implementation	7.83	1.45	2	8.3	.93	2
3. Methods for ensuring that behavioral interventions are implementable into practice and policy	7.75	1.52	4	8.21	.90	3
4. Use of systematic approaches to move from evidence to intervention components	7.9	1.19	1	8.11	.98	4
5. Selecting appropriate behavioral outcomes for trials	7.66	1.23	6	8.04	.68	5
6. Tailoring interventions to specific populations and contexts	7.69	1.66	5	7.96	1.20	6
7. Reporting intervention and comparison group(s) intervention content	7.64	1.26	7	7.93	0.92	7
8. Selection of suitable comparison group(s) within trials	7.55	1.15	8	7.86	0.85	8
9. Development of novel research designs to test behavioral interventions as alternatives to, or to complement, standard randomized controlled trials (RCTs)	7.42	1.46	11	7.60	1.25	9
10. Measurement of process(es) of change or mechanisms of action within interventions	7.44	1.53	10	7.56	1.38	10
11. Strategies to optimize intervention fidelity (including adherence)	7.29	1.43	13	7.53	1.09	11
12. Using theory in behavioral intervention development	7.04	1.8	18	7.49	1.35	12
13. Standardizing methods for reporting behavioral trials	7.25	1.49	15	7.47	1.31	13
14. Determining clinically significant changes in outcomes within trials	7.27	1.38	14	7.44	1.21	14
15. Relationship between behavioral outcomes and clinical/other outcomes	7.45	1.42	9	7.4	1.07	15
16. Standardized methods for reporting and registering behavioral trials’ protocols	7.16	1.58	16	7.35	1.38	16
17. Methods to assess intervention fidelity	7.27	1.64	14	7.3	1.38	17
18. Exploring impact of mode of intervention delivery	7.36	1.33	12	7.19	1.08	18
19. Strategies to maximize trial participant recruitment and retention	7.05	1.75	17	7.11	1.13	19
20. Investigating the impact of intervention intensity on outcomes (new item in survey 2)	N/A	N/A	N/A	7.11	1.18	19
21. Incorporating stakeholder input in intervention development and delivery	6.91	1.48	19	7.09	1.15	20
22. How to optimize stakeholder engagement in behavioral trial research	6.86	1.41	21	7.07	.94	21
23. Determining ideal timing of outcome measurement within trials	6.88	1.49	20	6.95	1.06	22
24. Novel approaches and designs for piloting behavioral interventions	6.86	1.88	21	6.91	1.47	23
25. Establishing criteria for progressing from trial piloting phases to full RCT	6.91	1.61	19	6.89	1.35	24
26. Contamination between study arms (intervention and comparison) within trials	6.84	1.57	22	6.79	1.24	25
27. Testing the impact of stakeholder engagement in behavioral trial research	6.70	1.74	23	6.70	1.36	26
28. Impact on intervention delivery of characteristics (such as qualifications and training) of those delivering interventions	6.56	1.57	24	6.67	1.26	27
29. Engaging stakeholders in the selection of outcomes (New item in survey 2)	N/A	N/A	N/A	6.63	1.54	28
30. Methods for cost-effectiveness analyses for behavioral trials	6.43	1.56	26	6.30	1.22	29
31. Trials’ research to test and develop behavioral theories	6.45	1.70	25	6.18	1.34	30
32. Strategies for handling missing data within behavioral trials	6.26	1.80	27	6.12	1.44	31
33. Developing novel statistical techniques to enhance behavioral trials	6.03	1.69	29	6.04	1.15	32
34. Blinding of researchers and participants to study arm allocation	6.1	1.90	28	5.93	1.50	33
35. Sample-size calculations for pilot studies	5.84	2.30	30	5.49	1.96	34

Two new items were generated by suggestions made by survey-1 respondents: Investigating the impact of intervention intensity on outcomes and Engaging stakeholders in the selection of outcomes. Therefore, participants received a list of 35 items to rate and rank in survey 2. Neither of these items scored above the median in survey 2: Investigating the impact of intervention intensity on outcomes received a mean rating of 7.11 (SD 1.18), putting it in 19th place of the 35 items; Engaging stakeholders in the selection of outcomes received a mean rating of 6.63 (SD 1.540, putting it in 28th place of the 35 items.

The number and percentage of participants who ranked each item as their top priority in surveys 1 and 2 are shown in Table 4. As in the item ratings, there were high levels of similarity in the items ranked most highly in surveys 1 and 2. The three items most frequently ranked as top priority in survey 2 were: Tailoring interventions to specific populations and contexts; Methods for ensuring that behavioral interventions are implementable into practice and policy; and Development of novel research designs to test behavioral interventions as alternatives to, or to complement, standard randomized controlled trials (RCTs). As with the item-importance ratings, the first and second items are from item categories Intervention development and Implementation. A new item appeared in the top-five priority items ranking as important, at number three, which was within the category Novel Trial Designs: Development of novel research designs to test behavioral interventions as alternatives to, or to complement, standard RCTs.

**Table 4:** Number and percentage of participants who ranked each item as their top priority in surveys 1 and 2, listed in order of the items that were most often selected as the top priority in survey 2

Research items	Survey 1		Survey 2	
	*N*	%	*n*	%
Tailoring interventions to specific populations and contexts	7	9.1	13	22.8
Methods for ensuring that behavioral interventions are implementable into practice and policy	6	7.8	8	14.0
Development of novel research designs to test behavioral interventions as alternatives to, or to complement, standard RCTs	6	7.8	6	10.5
Use of systematic approaches to move from evidence to intervention components	6	7.8	5	8.8
Determining clinically significant changes in outcomes within trials	2	2.6	5	8.8
Using theory in behavioral intervention development	10	13	4	7.0
How to disseminate behavioral trial research findings to increase implementation	4	5.2	2	3.5
Selection of suitable comparison group(s) within trials	5	6.5	2	3.5
Standardizing methods for reporting behavioral trials	0	0	2	3.5
Standardized methods for reporting and registering behavioral trials’ protocols	3	3.9	2	3.5
Specifying intervention components	7	9.1	2	3.5
Reporting intervention and comparison group(s) intervention content	1	1.3	1	1.8
Measurement of process(es) of change or mechanisms of action within interventions	2	2.6	1	1.8
Relationship between behavioral outcomes and clinical/other outcomes	1	1.3	1	1.8
Methods to assess intervention fidelity	1	1.3	1	1.8
Strategies to maximize trial participant recruitment and retention	3	3.9	1	1.8
Engaging stakeholders in the selection of outcomes (New item in survey 2)	n/a	n/a	1	1.8
Strategies to optimize intervention fidelity (including adherence)	1	1.3	0	0
Exploring impact of mode of intervention delivery	1	1.3	0	0
Investigating the impact of intervention intensity on outcomes (New item in survey 2)	n/a	n/a	0	0
Incorporating stakeholder input in intervention development and delivery	0	0	0	0
How to optimize stakeholder engagement in behavioral trials’ research	1	1.3	0	0
Determining ideal timing of outcome measurement within trials	0	0	0	0
Novel approaches and designs for piloting behavioral interventions	2	2.6	0	0
Establishing criteria for progressing from trial piloting phases to full RCT	2	2.6	0	0
Contamination between study arms (intervention and comparison) within trials	0	0	0	0
Testing the impact of stakeholder engagement in behavioral trial research	0	0	0	0
Impact on intervention delivery of characteristics (such as qualifications and training) of those delivering interventions	2	2.6	0	0
Methods for cost-effectiveness analyses for behavioral trials	0	0	0	0
Trials’ research to test and develop behavioral theories	0	0	0	0
Strategies for handling missing data within behavioral trials	0	0	0	0
Developing novel statistical techniques to enhance behavioral trials	0	0	0	0
Blinding of researchers and participants to study-arm allocation	0	0	0	0
Sample-size calculations for pilot	0	0	0	0
Selecting appropriate behavioral outcomes for trials	4	5.2	0	0

When respondents’ top-five priorities were given a weight and each item allocated a “ranking weighted score,” the top-five ranked items in surveys 1 and 2 were the same items, although the order changed slightly. Scores can be seen in Table 5. These were (in order of descending priority from the most highly ranking, weighted, scoring item from the rankings in survey 2): Tailoring interventions to specific populations and contexts; Methods for ensuring that behavioral interventions are implementable into practice and policy; Specifying intervention components; Use of systematic approaches to move from evidence to intervention components; and Development of novel research designs to test behavioral interventions as alternatives to, or to complement, standard RCTs. Again, the four highest scoring items in both surveys 1 and 2 were from categories: Intervention development and Implementation.

**Table 5:** Weighted ranking of participant responses to the “top-five” priorities question order by the most highly ranked item in survey 2

Item name	Weighted ranking score Survey 1	Overall rank Survey 1	Weighted ranking score Survey 2	Overall rank Survey 2
Tailoring interventions to specific populations and contexts	94	1	109	1
Methods for ensuring that behavioral interventions are implementable into practice and policy	72	5	97	2
Specifying intervention components	80	2	75	3
Use of systematic approaches to move from evidence to intervention components	72	5	73	4
Development of novel research designs to test behavioral interventions as alternatives to, or to complement, standard randomized controlled trials (RCTs)	74	4	67	5
How to disseminate behavioral trial research findings to increase implementation	75	3	63	6
Using theory in behavioral intervention development	70	6	57	7
Measurement of process(es) of change or mechanisms of action within interventions	59	7	57	7
Determining clinically significant changes in outcomes within trials	48	9	43	8
Selection of suitable comparison group(s) within trials	49	8	33	9
Reporting intervention and comparison group(s) intervention content	37	13	25	10
Selecting appropriate behavioral outcomes for trials	44	12	20	11
Standardizing methods for reporting behavioral trials	11	23	18	12
Methods to assess intervention fidelity	32	14	17	13
Strategies to maximize trial participant recruitment and retention	47	10	16	14
Strategies to optimize intervention fidelity (including adherence)	22	18	13	15
Engaging stakeholders in the selection of outcomes (New item in survey 2)	N/A	n/a	13	15
Relationship between behavioral outcomes and clinical/other outcomes	32	14	12	16
Standardized methods for reporting and registering behavioral trials’ protocols	29	15	10	17
Novel approaches and designs for piloting behavioral interventions	45	11	9	18
Establishing criteria for progressing from trial piloting phases to full RCT	27	16	8	19
Incorporating stakeholder input in intervention development and delivery	8	24	7	20
How to optimize stakeholder engagement in behavioral trials’ research	12	22	4	21
Impact on intervention delivery of characteristics (such as qualifications and training) of those delivering interventions	20	19	4	21
Methods for cost-effectiveness analyses for behavioral trials	18	20	3	22
Strategies for handling missing data within behavioral trials	3	27	2	23
Sample-size calculations for pilot	7	25	0	24
Developing novel statistical techniques to enhance behavioral trials	3	27	0	24
Determining ideal timing of outcome measurement within trials	8	24	0	24
Testing the impact of stakeholder engagement in behavioral trial research	6	26	0	24
Trials’ research to test and develop behavioral theories	14	21	0	24
Investigating the impact of intervention intensity on outcomes (New item in survey 2)	N/A	n/a	0	24
Exploring impact of mode of intervention delivery	23	17	0	24
Contamination between study arms (intervention and comparison) within trials	14	21	0	24
Blinding of researchers and participants to study-arm allocation	0	28	0	24

NB. Weights were calculated as follows: first priority = 5; second priority = 4; third priority = 3; fourth priority = 2; fifth priority = 1

## Discussion

### Summary of findings

The aim of this study was to identify priorities for methodology research specific to trials of behavioral interventions, and to seek the views of, and achieve consensus from, an international community of researchers working in this field. A large number of items was generated by the nine experts and many items from the long-list of 33 items were strongly endorsed as important methodological issues for behavioral trials’ research. There were no major changes between responses in survey 1 and responses in survey 2. From item-ratings and -rankings in both surveys, there was consensus around the types of items considered as most important or of highest priority. The four most highly rated items in terms of importance, in both surveys 1 and 2, came from two of the thematic categories, highlighting consensus that these are important priority areas for future methodological research within behavioral trials: Intervention development (Specifying intervention components and Use of systematic approaches to move from evidence to intervention components) and Implementation (How to disseminate behavioral trial research findings to increase implementation and Methods for ensuring that behavioral interventions are implementable into practice and policy). These items reasserted themselves as priorities from respondents’ ranking of their top-five priorities, with one new item emerging in the ranking, which had not been highlighted in the importance ratings: Development of novel research designs to test behavioral interventions as alternatives to, or to complement, standard RCTs.

Methodological challenges associated with the *development* of behavioral interventions were consistently identified as priorities within this study. These included, specifically, the challenges associated with specifying intervention components and the use of systematic approaches to move from evidence to intervention components. There has been significant recent progress in classifying the active components of behavior change interventions and methodological advances in the development of behavior change interventions. Replicable methods for identifying and reporting the active ingredients of behavioral interventions have been recently developed, including the Template for intervention description and replication (TIDieR) Checklist and Guide [17], the taxonomy of Behavior Change Methods [22] and the Behavior Change Technique (BCT) Taxonomy [34]. The BCT taxonomy has been widely adopted within health psychology; it provides an extensive, consensually agreed hierarchically structured taxonomy of 93 BCTs used in behavior change interventions.

In addition, frameworks have been developed to support the process of systematically moving from behavioral theory to intervention content. For example, Intervention Mapping [11], the Theoretical Domains Framework [6] and the Behavior Change Wheel [31] are all frameworks developed to support this process. While there has been rapid uptake of these tools since their publication, it is still early days to determine their impact on the quality and outcomes of behavioral intervention research and difficulties remain. For example, the process of identifying BCTs from behavioral interventions is not straightforward [19]. There is a lack of reliable methods for identifying which specific BCTs or BCT combinations have the potential to be effective for a given behavior in a given context [36]. The priorities identified in the current study reinforce the need for future work to focus on improving the reliability and robustness of descriptions of behavioral intervention components, and ensuring that during intervention development the active contents of interventions can be linked to the theoretical premises for behavior change. These issues are central to an ongoing program of research called the “Human Behavior-Change Project,” where behavioral scientists are working with computer scientists to develop an online knowledge system (an ontology) to facilitate the identification, extraction and synthesis of knowledge related to behavior change interventions [32, 40].

The identification of the methodological research priority “Development of novel research designs to test behavioral interventions as alternatives to, or to complement, standard randomized controlled trials (RCTs)” may assist in resolving some of the challenges identified above in the development and specification of theory-based interventions. There has been a growing interest within behavioral science in novel research designs that can provide information beyond that provided by the standard RCT design. The classic, two-armed RCT allows us to test the effectiveness of one intervention *package* compared to another intervention *package*. However, this design is of limited use to inform our understanding of the relative importance or potency of constituent intervention components, the optimal dose of each component, the optimal combination or sequence of delivery of components, or their mechanisms of action to effect behavior change [7]. There is a growing number of studies in the literature leveraging alternative frameworks and trial designs such as the Multiphase Optimization Strategy (MOST) and the Sequential Multiple Assignment Randomized Trial (SMART) design. The Multiphase Optimization Strategy (MOST) is an engineering-inspired methodological framework for optimizing and evaluating interventions [7]. MOST uses randomized experimentation to assess the performance of individual intervention components and their interactions in an optimization trial, to optimize interventions in advance of testing through RCTs. MOST has been used in a number of settings, including to optimize interventions in Internet cognitive-behavioral therapy for depression [50], human immunodeficiency virus (HIV) care [16], smoking cessation [44] and remotely delivered intensive lifestyle treatment for obesity [42]. The SMART design allows evaluation of adaptive interventions in which the type or dose of treatment is individually tailored based on the patient’s needs [2, 37]. A SMART design has been used in a number of areas; for example, to evaluate alternative combinations of perinatal interventions and sequencing patterns to optimize women’s health outcomes [14]. These approaches are still in their infancy and behavioral scientists should use and develop these frameworks to enhance the quality of behavioral intervention research.

There is potential for digital health-behavior change interventions to enhance our understanding of behavior change mechanisms [38] and enable more sophisticated research designs which promote a more nuanced understanding of intervention processes. For example, the just-in-time adaptive intervention (JITAI) is an intervention design developed within digital health intervention research which aims to provide the right type and amount of support, at the right time, by adapting to an individual’s changing internal and contextual state [39]. Increasingly powerful mobile and sensing technologies within JITAIs enable the monitoring of changes to an individuals’ state and tailored delivery of intervention components. Research on the development and evaluation of these interventions is still very limited and it is critical that researchers develop sophisticated and nuanced health behavior theories capable of guiding the construction of such interventions in line with the rapidly growing technological capabilities for delivering JITAIs.

In addition, qualitative research should be used more comprehensively within behaviour change intervention research to enhance quality. Qualitative research can enhance pre-trial intervention development and strengthen the interpretation of the findings of intervention trials by shedding light on implementation issues and understanding the impact of intervention context on effectiveness [41].

The other methodological research category identified as a high priority in this study, was the area of implementation. Gaps in methods to ensure translation of behavioral trial research findings into practice and policy were strongly endorsed as important by respondents in this study, as was the lack of strategies to effectively disseminate behavioral trial research findings to increase implementation. Difficulties in dissemination and implementation of research findings is not unique to behavioral trials; the gap between research evidence and routine practice has been identified as a consistent feature of health care delivery [27]. Integrated Knowledge Translation (IKT) has been suggested as a method to increase the relevance and applicability of research by engaging knowledge users through the entire research process, not just at the end of a project [15]. Indeed, stakeholder engagement, which refers to the involvement of public, patients, health professionals, service users, funders and other decision-makers in research, should be used throughout the whole research process to enhance the relevance, quality and impact of behavior change intervention research [4]. Exploring ways to incorporate emerging IKT methods within behavioral trials’ research may strengthen the potential impact of behavioral science research in improving health and health care.

### Strengths and limitations

This is the first study which has attempted to systematically achieve consensus on methodological research priorities for behavioral trials’ research. The study protocol was published on an open-access publication platform and was subjected to transparent peer review [5]. The study was conducted in line with internationally recognized guidelines for the Conducting and Reporting of DElphi Studies (CREDES) [21].

Caution is needed in generalizing the findings, as the response rate for survey 1 (25%) was relatively low compared with other research prioritization e-Delphi studies (for example, [9] achieved a 42% response rate to survey 1 in their study). However, the retention rate for participants in survey 2 was adequate (74%). The sampling frame was limited to members of the IBTN and the sample of the e-Delphi survey was relatively small. Responders may have differed from non-responders; we did not have data on the full sampling frame to enable comparison. While we achieved a reasonable spread of countries internationally in the sample, respondents are drawn largely from developed countries. Developing countries are not represented. Methodological challenges associated with behavior change intervention research in developing countries are likely to differ significantly from those relevant in developed countries [1]. The majority of IBTN members and participants in this study are researchers, with academic appointments or are in graduate training programs. Health professionals, policy-makers, patients and the public were underrepresented or absent from the study. It would be useful to obtain the views of more diverse stakeholder groups in future research.

A further potential limitation to note in relation to the Delphi process was that members of the research team (MB, JMS, KL and SB) were also members of the expert panel that generated the initial long-list of items. This was done as we wanted to maximize the number of items generated for the long-list. However, this may have been a source of bias in the initial process of refining the list of items for the survey.

## Conclusion

Given the significant potential impact of behavioral interventions on global health, ensuring that we are conducting high-quality research is imperative. While caution is needed in interpreting the findings of this study due to the relatively low response rate and small sample size, the priorities identified in this study can be used to inform the research agenda of the IBTN and could be used more broadly to inform the behavioral trials’ methodology agenda internationally. Furthermore, the results of this study can be leveraged by national and international funding bodies to help identify and shape resource allocation, and could be used to advocate for targeted research calls. Specifically, future research should prioritize: improving strategies to systematically develop interventions and specify intervention components; exploring novel research designs which allow us to develop more effective interventions and better understand what intervention components work for whom in what settings; and developing strategies to ensure that the findings from behavioral intervention research can be translated into practice and policy.

## Data Availability

The datasets used and/or analyzed during the current study are available from the corresponding author on reasonable request.
